# Basketball Fatigue Impact on Kinematic Parameters and 3-Point Shooting Accuracy: Insights across Players’ Positions and Cardiorespiratory Fitness Associations of High-Level Players

**DOI:** 10.3390/sports12030063

**Published:** 2024-02-20

**Authors:** Dimitrios I. Bourdas, Antonios K. Travlos, Athanasios Souglis, Dimitrios C. Gofas, Dimitrios Stavropoulos, Panteleimon Bakirtzoglou

**Affiliations:** 1 Section of Sport Medicine & Biology of Exercise, School of Physical Education and Sports Science, National and Kapodistrian University of Athens, 41 Ethnikis Antistasis, 17237 Daphne, Greece; dbourdas@phed.uoa.gr (D.I.B.); asouglis@phed.uoa.gr (A.S.); 2 Department of Sports Organization and Management, Faculty of Human Movement and Quality of Life Sciences, University of Peloponnese, Efstathiou and Stamatikis Valioti & Plataion Avenue, 23100 Sparta, Greece; atravlos@uop.gr; 3 Arsakeia-Tositseia Schools, Philekpaideftiki Etaireia, Mitilinis 26, 11256 Athens, Greece; gofasd@hotmail.com; 4 School of Physical Education and Sport Science, Aristotle University of Thessaloniki, University Campus, 54124 Thessaloniki, Greece; dnstavro@phed.auth.gr; 5 Faculty of Sport Sciences & Physical Education, Metropolitan College, Eleftheriou Venizelou 14, 54624 Thessaloniki, Greece

**Keywords:** aerobic–anaerobic, exertion, fatigue, high-intensity exercise, metabolism, muscle function, performance, ventilatory threshold, RPE, velocity

## Abstract

This study investigated the impact of basketball-induced fatigue on 3-point jump shooting accuracy, the ball’s entry angle (EA) into the hoop, shot release time (RT), their relationship with player positions in high-level basketball, and the correlation between cardiorespiratory fitness markers and potential shooting performance changes. Guards (n = 13), forwards (n = 13), and centers (n = 12) underwent physiological assessments. Sequentially, they performed 15 jump shots (PRE), a basketball exercise simulation (BEST) involving 24 × 30 s circuit activities, and a repeated shooting test (POST). The study design was double-blind. The results revealed significant differences (*p* ≤ 0.05) in RT, EA, and successful shots (SSs) between PRE and POST in each group. The percentage changes from PRE to POST conditions across guards, forwards, and centers were for RT: 25.34% [95%CI: 1.7–48.98], 19.73% [95%CI: −1.9–41.36], 14.95% [95%CI: −5.23–35.13]; for EA: −3.89% [95%CI: −14.82–7.04], −3.13% [95%CI: −12.9–6.64], −3.47% [95%CI: −14.19–7.25]; and for SS: −14.42% [95%CI: −36.5–7.66], −16.76% [95%CI: −40.81–7.29], −19.44% [95%CI: −46.7–7.82], respectively. Post-test differences (*p* ≤ 0.05) highlighted greater fatigue impact on RT, EA, and SS from guards to centers. Additionally, significant correlations (*p* ≤ 0.05) were found between the ventilatory threshold, mean HR during BEST, and changes in RT, EA, and SS. This study highlights the substantial impact of basketball-induced fatigue on 3-point shooting parameters across player positions and the interplay with cardiorespiratory factors post-fatigue. Tailored training, considering heart rate, is crucial to optimizing shooting performance.

## 1. Introduction

The significance of shooting in basketball cannot be overstated, as winning a game heavily relies on successful shots (SSs) [1]. Among various shooting techniques [2] the jump shot stands out, constituting over 40% of total points in a basketball game [3,4]. The jump shot is a fundamental shooting technique where a player propels themselves off the ground (jumping vertically) while simultaneously releasing the ball (near the peak of the jump to maximize accuracy and distance) toward the hoop in an attempt to score points [5,6]. This type of shot involves a coordinated movement of the legs, torso, and arms to generate power and precision in the shot’s trajectory [5,6]. It is a versatile technique, with variations in release angles, timing, and elevation depending on the shooter’s position and defensive pressure [5,6].

The angle at which a basketball is released is widely regarded as a crucial determinant for the effectiveness of a jump shot [7,8,9]. A higher release angle correlates with a higher trajectory of the basketball, facilitating an increased entry angle (EA) into the hoop [7,8,9]. This heightened EA (closer to perpendicular) optimizes the utilization of the basket’s rim area to enhance the ball’s trajectory through the hoop, necessitating a greater release velocity for successful execution [7,8,9]. However, while the EA is interconnected with the release angle, this relationship is subject to variations dictated by multiple factors, such as the distance between the shooter and the basketball hoop [10], the height of the release, and the potential defensive pressure exerted by an opposing player [4]. In practice, the ideal EA for a successful jump shot typically falls within the range of approximately 42° to 48° [11], with the optimal angle observed around 45° [8,9]. Beyond the EA into the hoop, the ball’s release time (RT) in a jump shot (i.e., the duration from the instance the player receives the ball to the moment it departs from their hand during the shooting action) stands as a critical parameter, influencing the swiftness and efficiency of the shot. Studies have indicated that elite senior Serbian basketball players, categorized as guards, forwards, and centers, exhibit varying total RT for a 3-point jump shot, ranging approximately between 0.76 and 0.83 s, and for a 2-point jump shot, ranging approximately between 1.07 and 1.23 s [12,13]. Therefore, as perceived, these particular kinematic factors, the EA and the RT, have been proposed as primary factors affecting the accuracy of jump shots, particularly as shooting distances from the hoop increase, influencing shooting performance [10].

Contemporary basketball demands a high level of proficiency in technical skills [2,14,15]. From a physiological standpoint, basketball represents an intermittent sport characterized by a mix of high-intensity, short-duration actions (2–5 s) such as jumping, sliding, and sprinting, alongside prolonged (~5–15 s) lower-intensity activities like walking and running [16]. As a consequence (excluding the psychological pressure of the competitive basketball game [17]), the physiological demands on players during a basketball game are substantial, necessitating the utilization of both aerobic and anaerobic energy systems [18,19]. Various studies have indicated that players often exhibit near-maximal values in parameters such as blood lactate (La^−^) levels, mean heart rates (HRs), and oxygen uptake during competitive games [16,18,20,21]. This proximity to maximal physiological limits suggests a potential impact of fatigue on player physical performance, coordination, and technical skills, potentially leading to a decline in game efficiency [22]. However, only a few studies have delved into the impact of fatigue on shooting accuracy and various kinematic parameters associated with basketball shooting, such as the ball’s flight trajectory, joint angles in the upper and lower extremities, and the center of mass during shooting [23,24,25,26,27,28]. Moreover, the fatigue induction protocols employed in the aforementioned studies lacked significant alignment with the conditions experienced in a competitive basketball game, thereby presenting a potential disparity in the controlled assessment of kinematic parameters. There is also a paucity of research investigating the effects of fatigue on shooting accuracy, EA into the hoop, and jump shot RT in basketball, further contributing to the existing knowledge gap in this domain. Consequently, the effects of basketball-induced fatigue on shooting accuracy, specific shooting kinematics (i.e., EA into the hoop and jump shot RT), and their correlation with player positions in basketball remain predominantly unexplored. On the other hand, athletes with higher levels of cardiorespiratory fitness displayed prolonged resistance to fatigue and demonstrated enhanced accuracy and consistency in free throw shooting under fatigue conditions [29]. Nevertheless, a distinct research gap persists concerning the exploration of how players’ cardiorespiratory fitness correlates with accuracy in jump shooting and associated kinematic parameters under conditions of fatigue.

Jump shots are used for both 2-point and 3-point shots. The EuroLeague records approximately 35–37 attempted 2-point shots per game, encompassing all 2-point shot types such as layups, set shots, and jump shots [17]. However, over recent years a prevailing notion suggests a transformative phase in the NBA—a ‘3-point revolution’—marked by a consistent increase in 3-point jump shot attempts throughout the seasons [30]. Similarly, the EuroLeague witnessed around 22–23 attempted 3-point jump shots per game during the 2019–2021 seasons [17]. Hence, optimizing success rates for 3-point jump shots has emerged as a pivotal factor influencing game outcomes [17,30].

In summary, the significance of 3-point jump shots in contemporary basketball is evident in their impact on game outcomes; the ball’s EA into the hoop and jump shot RT are considered to be the main elements that influence 3-point jump shot performance. However, research to examine the effects of fatigue on 3-point shooting performance is still scarce [31,32,33], particularly among high-level players. Additionally, the investigation of kinematic parameters like EA into the hoop and jump shot RT concerning player positions is notably lacking in the current research landscape. Hence, considering the aforementioned context, this study aimed to explore the effects of fatigue induced by basketball activity on 3-point jump shooting accuracy, entry angle into the hoop, jump shot release time, and their potential associations with player positions in high-level basketball players. Our main hypothesis was centered on the notion that fatigue induced by basketball activities would adversely affect both 3-point jump shooting accuracy and associated kinematic parameters. Furthermore, this study aimed to explore the relationship between cardiorespiratory fitness markers and potential changes in shooting performance.

## 2. Materials and Methods

### 2.1. Study Population

In this investigation, we assessed a sample comprising 38 high-level basketball athletes sourced from the top three Greek national basketball leagues. These participants were categorized into three distinct groups based on their designated playing positions: guards (N = 13), forwards (N = 13), and centers (N = 12). Comprehensive anthropometric and physiological profiles for each of these groupings are presented in Table 1. The inclusion criteria for participants necessitated a minimum of six years of active engagement in competitive basketball, an age threshold of 18 years or older, and a consistent commitment to maintaining a specific level of physical activity, defined as no less than a daily dose of moderate-to-vigorous physical activity, equivalent to a minimum of 60 min per day [34].

**Figure 1:**
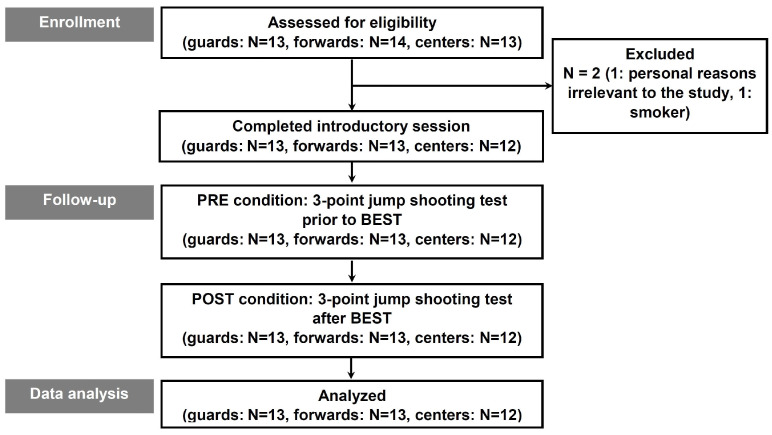
Schematic representation of the study’s experimental framework. Abbreviations: BEST, basketball exercise simulation test; N, sample size of the groups.

In contrast, we established exclusion criteria for prospective participants, which involved the exclusion of individuals who had reported musculoskeletal injuries occurring at least six months prior to the commencement of the study, the presence of significant respiratory, cardiovascular, or other severe medical conditions, active medication regimens, a history of smoking or the use of nicotine-related products, or a consistent daily sleep duration of less than 8 h. To assess the participants’ self-reported physical activity levels and overall well-being we employed the Active-Q and PAR-Q+ questionnaires [35,36], respectively. Furthermore, participants provided information on their smoking habits and their perceived sleep adequacy as per the guidelines provided in reference [37]. Ethical approval for the study was granted by the Institutional Review Board at the local university, under protocol number 3447. We obtained written informed consent from all study participants, providing them with comprehensive information about the study’s laboratory and field conditions, the methodologies employed, as well as potential risks involved, all in strict adherence to the latest ethical guidelines outlined in the Helsinki Declaration [38]. A visual representation of the study’s research design is depicted in Figure 1. All participants refrained from the consumption of beverages or medications containing caffeine or alcohol, were in good overall health, reported a sense of well-being, resided at altitudes below 1500 m, and did not engage in blood donation activities during the course of the experimental procedures.

### 2.2. Initial Assessments

At the commencement of the present study, during the initial visit to the research facility, all enrolled participants received a thorough familiarization with both the laboratory and the court-field facilities. They received a detailed introduction to the specific research methodologies that would be employed in this investigation. Following that, measurements encompassing height (Stadiometer^®^, Seca, Birmingham, UK), body mass (Beam Balance 710, Seca, Birmingham, UK), and body fat were assessed through specific equations based on seven skinfold measurements (chest, axilla, triceps, subscapula, abdomen, supra-iliac, and thigh) using Harpenden skinfold calipers (Baty International, West Sussex, UK) within the study population [39], along with countermovement jump (CMJ) height, incorporating an arm swing element on a contact platform (EuroJump^®^, Newtest, Oulu, Finland) were conducted (for additional details, see the online Supplementary Methods File) [40]. Subsequently, a maximal oxygen uptake ($\dot{V}$O_2max_) assessment was performed using a calibrated metabolic cart (Vacumed Mini-CPX, Ventura, CA, USA) during an incremental treadmill exercise test to exhaustion conducted on a Technogym Runrace treadmill (Technogym, Gambettola, Italy). The protocol began with a 1 min run at 7 km·h^−1^, followed by a 30 s increase to 8 km·h^−1^. The treadmill speed was subsequently incremented by 0.5 km·h^−1^ every 30 s until exhaustion, maintaining a 1% incline. The $\dot{V}$O_2max_ and maximum heart rate (HR_max_) were determined as the peak values observed within 15 s and 5 s, respectively, during the final exercise phase. The criteria for achieving $\dot{V}$O_2max_ included meeting at least two of the following: a respiratory exchange ratio surpassing 1.1, HR_max_ aligning within 10 b·min^−1^ of the estimated age-based HR_max_, a rating of perceived exertion (RPE) of 18 or higher, or a $\dot{V}$O_2max_ plateau < 2 mL·kg^−1^·min^−1^ with a simultaneous treadmill speed increase [41]. Telemetric heart rate monitoring (Polar RCX5, Polar Electro Oy, Kempele, Finland) and the Borg 6–20 linear scale assessed the subjective rating of perceived exertion [42]. Furthermore, the second ventilatory threshold (VT_2_) was determined based on the exercise intensity test, where the plot depicting the minute production of CO_2_ against the minute utilization of oxygen ($\dot{V}$O_2_) exhibits an increase in slope exceeding 1.0 [43,44,45]. The estimation of VT_2_ was conducted through consensus by two independent blinded specialists, and the averaged determination was retained for subsequent analysis, expressed as a percentage of $\dot{V}$O_2max_.

**Figure 2:**
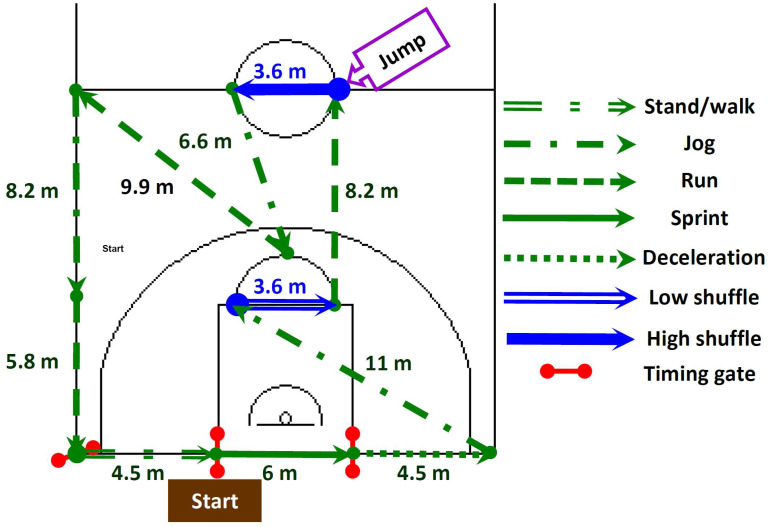
Description of the basketball exercise simulation test (BEST) as per reference [47].

**Figure 3:**
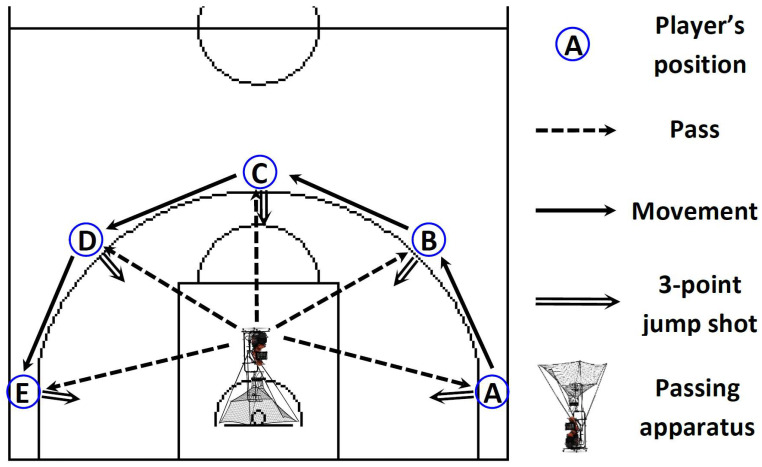
The 3-point shooting test. The participants performed a sequence of three consecutive 3-point jump shots from the A to E positions (i.e., left corner, left wing, top, right wing, and right corner), 15 shots in total.

After a recovery period of approximately 30 min following the $\dot{V}$O_2max_ test, participants engaged in a structured familiarization process with the basketball exercise simulation test (BEST) protocol [46], as visually depicted in Figure 2. This familiarization process included participants practicing the BEST at different intensities, culminating in approximately 6–8 circuits performed at maximal intensity, as per established protocol [46]. Next, participants were familiarized with a basketball 3-point shooting test (Figure 3).

### 2.3. Methodological Protocols

Seven days later, in the second visit, participants were instructed to perform a basketball shooting test (PRE condition), undergo the BEST, and then execute one more basketball shooting test (POST condition), with each set of basketball shooting tests separated by 60 s intervals from the BEST. These activities took place on a standard indoor hardwood basketball court, and participants were specifically instructed to exert maximal effort in accordance with their customary practice methods.

In the two days leading up to both the first and second (laboratory and field) visits, participants adhered to a regimen of avoiding rigorous physical activities. They exclusively engaged in post-game recovery training sessions focused on reinforcing standard game strategies and promoting team cohesion [48,49]. Additionally, participants refrained from using any supplements that might possess ergogenic or synergistic properties, consistent with prior research findings [50,51,52]. Furthermore, on the evening before each experimental session, participants were provided with a standardized dinner [53]. In specific reference to the second visit, participants arrived at the basketball court between 9:00 and 9:30 a.m. following an overnight fasting period. Throughout all experimental sessions, the basketball court was maintained at uniform environmental conditions, featuring an approximate air temperature of 23–25 °C, barometric pressure ranging from 1010 to 1030 mmHg, and a consistent relative humidity level of approximately 50%.

Before initiating any experimental procedures, participants were instructed to empty their bladders. Prior to engaging in any performance-based evaluations, participants completed a standardized 20 min warm-up routine. This warm-up routine consisted of activities such as low-intensity jogging, dynamic full-body stretches, brief intervals of high-intensity running, and, in the second visit, fifty 3-point jump shots. Furthermore, preceding each assessment, all measuring instruments underwent calibration procedures in strict accordance with the manufacturer’s provided specifications.

Participants were intentionally kept unaware of their shooting performance and their results in the BEST. They were also discouraged from discussing the study with others to avoid introducing any expectations, whether positive or negative, until the study’s conclusion. Furthermore, both participants and assisting researchers were blinded to the true objective of the study. The study was carried out during the first phase of the regular season in December, with the presumption that participants had not yet accumulated substantial fatigue from an extensive series of matches.

### 2.4. Field Basketball Exercise Simulation Test (BEST)

The BEST (Figure 2) comprised a series of 30 s circuit-based activities involving various movement distances (low-intensity activity, constituting 727 m (42% of the total distance), encompassing standing, walking, and jogging; high-intensity activity, accounting for 826 m (48% of the total distance), including running and sprinting; and shuffling activity, representing 172 m (10% of the total distance)), as previously detailed in reference [46]. Participants engaged in these circuits continuously for a total duration of 12 min, aiming to complete a maximum of 24 circuits within the designated timeframe. In cases where participants were unable to complete a circuit within the 30 s interval, no rest was allowed, and they were immediately required to initiate the subsequent circuit. Consequently, participants in such instances did not attain the target number of 24 circuits during testing unless they successfully restored the standard circuit timing. 

The recording of circuit and sprint times (CT, ST) was meticulously conducted using infrared photoelectric cells positioned at a height of 1.1 m. These cells were seamlessly integrated into a timing system (Saint Wien Digital Timer Press H5K, Lu-Chou City, Taipei Hsien, Taiwan). Next, for assessing the fatigue index during the BEST [54], CT and ST decreases were evaluated as the cumulative percentage decline in circuit and sprint performance by analyzing the mean times gathered across each pair of circuit efforts. The decreases in the CT and ST were determined using the formula [((total time/ideal time) × 100) − 100], where the ideal time represented the fastest circuit and sprint interval, respectively [54]. Furthermore, to monitor HR data during the BEST we employed telemetric technology (Polar RCX5, Polar Electro Oy, Kempele, Finland), which collected HR measurements at 5 s intervals throughout the entire duration of the BEST. This facilitated the calculation of the average HR during the BEST. 

Our decision to employ the BEST as a basketball simulation physical performance test is underpinned by its alignment with the distances typically observed in competitive adult male basketball at elite and sub-elite levels, as detailed in reference [55]. In these competitive settings, the distribution of distances covered closely parallels the parameters of our chosen test, with low-intensity activity typically accounting for approximately 40–44% of the total distance, high-intensity activity representing approximately 47–51%, and shuffling activity constituting roughly 3–4% of the total distance. Additionally, it is worth noting that the BEST exhibits favorable attributes as a reliable and valid test specific to basketball [46]. This is evident in its substantial test–retest reliability, with robust intra-class correlation coefficients (ranging from 0.98 to 0.99) and coefficients of variation (ranging from 1.4% to 1.7%) for mean circuit and sprint times [46]. These findings underscore its suitability for assessing both anaerobic and aerobic fitness components pertinent to basketball performance.

### 2.5. Basketball Shooting Test and Kinematic Parameter Measurements

The participants, positioned in the offensive/triple-threat stance (with flexed ankle, knee, and hip joints; maintaining direct eye contact with the basketball hoop; standing with both feet positioned on the ground at shoulder width), performed a sequence of three consecutive 3-point jump shots from five distinct field positions (left corner, left wing, top, right wing, and right corner, 15 shots in total) with no defense pressure (Figure 3). Each participant was instructed to shoot as he would in a competitive game, directly through the hoop, and had a time limit of 90 s to complete the set of 15 shots. To facilitate this, we employed smart sensor basketballs (94Fifty Basketball, InfoMotion Sports Technologies Inc., Dublin, OH, USA) and an apparatus (Dr. Dish CT_+_, Airborne Athletics, Inc., Minneapolis, MN, USA) for collecting and passing the balls at the player’s chest level at the same intensity in predefined time intervals. The passing apparatus facilitated the documentation of SSs, allowing the capture of shooting accuracy and the subsequent calculation of shooting success rate (SSR) for later analytical purposes. The “smart” basketballs were designed to meet regulation size and weight standards and were equipped with nine individual pressure sensors, a Bluetooth chip, and an eight-hour wirelessly rechargeable battery. The strategically positioned sensors on the basketballs offered a comprehensive 360-degree assessment of the forces applied to the ball, enabling meticulous monitoring of all aspects of ball contact. The basketballs were wirelessly linked to the 94Fifty app, which offered real-time feedback based on data collected by the basketball’s sensors. This feedback encompassed parameters such as the ball’s EA and RT, with data transmission occurring within less than 100 milliseconds and extending over a range of up to 30 m. Previous studies have also substantiated the reliability and validity (α = 0.998) of these kinematic parameters concerning video analysis, underscoring their accuracy and consistency [56,57].

### 2.6. Other Measurements (Blood Lactate Concentration, Subjective Rating of Perceived Exertion, and Perceived Level of Muscle Soreness)

Fifteen minutes after the end of the BEST (and the execution of the basketball shooting test in the POST condition), various measurements were also conducted. Blood samples were obtained from the participants’ left fingertips, yielding 7 μL of capillary blood, and afterwards subjected to analysis (StatStrip^®^ Xpress™ Lactate Analyzer, Nova Biomedical, Waltham, MA, USA) to determine blood La^−^ levels [58]. Each blood sample underwent dual measurements, and the resulting values were averaged for subsequent statistical analysis. The manufacturer’s internal studies provided coefficients of variation for typical imprecision, covering both within-run and day-to-day variability (ranging from 3.4% to 5.9% for lactate values between 2.6 and 10.5 mmol·L^−1^) [59].

The evaluation of the subjective rating of perceived exertion (RPE) was conducted using the 6–20 Borg scale [33]. RPE, as a subjective measure, was employed to gauge the perceived level of overall muscular effort and the presence of fatigue. Furthermore, following the completion of three squat positions, each participant underwent a 3 s palpation, after which they were required to fill out a questionnaire to evaluate perceived muscle soreness. This questionnaire entailed participants rating the level of overall muscle soreness (RPMS) in specific leg muscles (i.e., knee extensor and flexor) for both legs. Participants provided ratings on a scale that ranged from 0 (indicating the absence of soreness) to 10 (indicating a high level of soreness) [60]. The mean levels of blood La^−^, RPE, and RPMS following the completion of the BEST, as well as the mean HR as a percentage of the maximum HR, CT, and ST decreases observed during the BEST, were utilized to assess the comparability of participant efforts and conditions across all groups before the execution of the basketball shooting test in the POST condition.

### 2.7. Statistical Analyses

The data met the assumptions of normality and homogeneity of variances, as indicated by Shapiro–Wilk tests (*p* > 0.05) and Levene’s tests (*p* > 0.05). To compare participants’ characteristic variables, the mean HR, CT, and ST decreases during the BEST, as well as variables related to La^−^, RPE, and RPMS after the BEST and before the basketball shooting test, among the different player groups (i.e., guards, forwards, and centers), one-way analysis of variance (ANOVA) for independent groups was applied. To compare the mean RT, EA, SS, and SSR variables within the same player groups (i.e., guards, forwards, and centers) and overall under the two conditions (i.e., PRE and POST), paired *t*-tests were performed [61]. One-way ANCOVA was also conducted to examine whether there were any significant differences in the POST condition’s mean RT, EA, SS, and SSR adjusted means (i.e., the PRE condition was used as a covariate) between the different player groups (i.e., guards, forwards, and centers). In cases of statistically significant differences, post hoc analysis (Bonferroni pairwise comparisons) was carried out [61]. Moreover, the Pearson correlation coefficient (r) was employed to assess the magnitude (i.e., small: r ≈ 0.10, medium: r ≈ 0.30, large r ≈ 0.50 [62]) and direction of the relationship among physiological parameters and the potential differences in RT, EA, and shooting accuracy between the POST and PRE conditions within the studied participant sample. Statistical analyses were executed using the SPSS software platform (version 29.0, IBM Corp., Armonk, NY, USA), and a significance level of α = 0.05 was adopted for all statistical tests. In addition, a post hoc power analysis was executed using the GPower 3.1.9.2 software (Heinrich-Heine-University, Düsseldorf, Germany). The analysis utilized RT, EA, and SS as the criterion variables, considering the following parameters: effect size, d = 1.00; significance level, (α) = 0.05; a sample size of 12; and a design involving one group with two conditions. The resulting observed power (1 − β) surpassed 0.90.

### 2.8. Preliminary Study

The SS during the BEST underwent reliability testing, involving two consecutive assessments on the same day with a 14 min interval within the same participant pool. A robust correlation coefficient between the repeated measures of SS (r(_36_) = 0.73, *p* ≤ 0.05) was established. Furthermore, the coefficient of variation (CV) for the mean Δ values between the two sets of measurements stood at 10.20%. The mean differences between the two performances were found to be within an acceptable range of –0.13 ± 0.74 (SD) based on the 95% confidence interval derived from the Bland–Altman plot [63]. The limits of agreement (–1.61 to 1.35 = ±1.96 SD) demonstrated a narrow range and the intraclass correlation (ICC) was between fair and excellent, being 0.84 (0.69 to 0.92), indicating a high level of reliability in assessing shooting accuracy during the BEST.

## 3. Results

**Figure 4:**
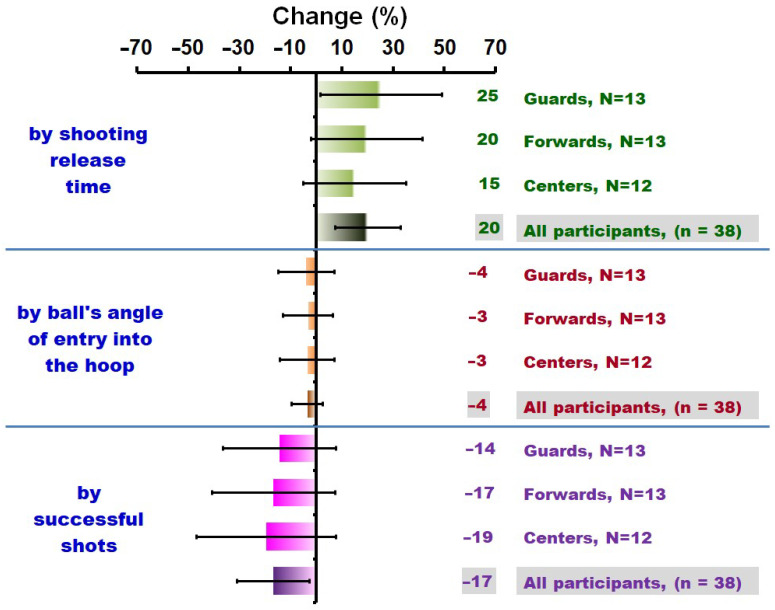
Changes in 3-point jump shot release time, ball’s entry angle into the hoop, and successful shots values (from the PRE to the POST conditions) across groups and in all participants. Error bars present the lower and upper bounds of the 95% confidence intervals. Abbreviations: BEST, basketball exercise simulation test; n, sample size of the study; N, sample size of the groups; POST, condition after the BEST; PRE, condition prior to the BEST.

All data are expressed as mean (M) ± standard deviation (SD) [95% confidence interval (CI)] unless otherwise specified. Table 1 outlines the descriptive characteristics of each group. The variables—ST decrease, CT decrease, mean HR during the BEST, La^−^, RPE, and RPMS—for both PRE and POST conditions across groups are presented in Table 2. A significant difference in ST decrease (F(_2,35_) = 8.467, *p* < 0.001) was noted, notably higher in the centers group (29.33 ± 1.40% [28.54–30.12]) compared to guards (27.84 ± 0.59% [27.52–28.16], *p* = 0.001) and forwards (28.16 ± 0.70% [27.78–28.54], *p* = 0.011) (Table 2). The assessment of the mean RT, EA, SS, and SSR revealed significant differences (all *p* < 0.05) between conditions (PRE and POST) within each group (Table 3). Figure 4 visually depicts the changes (%) in RT, EA, and SS from PRE to POST conditions among participants and all groups. Notably, the 95%CI for the change in RT ranged from 7.39% to 32.89% across all participants, from 1.70% to 48.98% among guards, from –1.90% to 41.36% among forwards, and from –5.23% to 35.13% among centers. Similarly, the 95%CI for the change in EA ranged from –9.55% to 2.55% across all participants, from –14.82% to 7.04% among guards, from –12.90% to 6.64% among forwards, and from –14.19% to 7.25% among centers. For SS, the 95%CI spanned from –30.90% to –2.72% across all participants, from –36.50% to 7.66% among guards, from –40.81% to 7.29% among forwards, and from –46.70% to 7.82% among centers.

Further statistical analysis utilizing one-way ANCOVA tests indicated statistical significance for RT (F(_2,34_) = 7.43, *p* < 0.002), EA (F(_2,34_) = 9.22, *p* < 0.001), and SS (F(_2,34_) = 8.35, *p* < 0.001). Subsequent post hoc analysis unveiled significant differences: (a) for RT, guards were faster (0.71 ± 0.03 s [0.64–0.78]) than forwards (0.84 ± 0.02 s [0.79–0.89]) and centers (0.95 ± 0.04 s [0.88–1.03]), and forwards were faster than centers; (b) for EA, guards achieved higher angles (43.41 ± 0.41° [42.58–44.23]) than forwards (41.97 ± 0.29° [41.38–42.56]) and centers (40.41 ± 0.41° [39.58–41.25]), and forwards achieved higher angles than centers; and (c) for SS, guards were more successful (6.64 ± 0.22 [6.19–7.09]) than forwards (5.92 ± 0.17 [5.58–6.27]) and centers (5.05 ± 0.24 [4.56–5.54]), and forwards were more successful than centers (Table 4). Additionally, Table 5 shows the Pearson correlation coefficients (r) for $\dot{V}$O_2max_, VT_2_, and mean HR during the BEST and observed differences in RT, EA, SS, and SSR between POST and PRE conditions within the studied sample. RTs were significantly correlated with VT_2_ (r(_36_) = –0.502, *p* < 0.002) and mean HR (r(_36_) = 0.75, *p* < 0.001), EAs with VT_2_ (r(_36_) = 0.48, *p* < 0.003) and mean HR (r(_36_) = –0.64, *p* < 0.001), and SSs with VT_2_ (r(_36_) = 0.50, *p* < 0.002) and mean HR (r(_36_) = –0.68, *p* < 0.001), indicating a large-magnitude relationship [62].

## 4. Discussion

The primary aim of this investigation was to explore the influence of fatigue induced by a basketball exercise simulation test on 3-point jump shooting accuracy, release time, and the entry angle of balls into the hoop, considering player positions in basketball. The results demonstrated a notable decrease in shooting accuracy, entry angles, and an increase in release time post-basketball simulation test. Moreover, the impact of fatigue on shooting accuracy and kinematic parameters was more pronounced from guards to centers. In addition, the interdependence of cardiorespiratory factors and shooting performance following fatigue was highlighted.

Contemporary basketball stands as a dynamic team sport [16], requiring a proficient command of technical skills [2,14,15] and imposing significant physiological demands on its players, calling for a blend of aerobic and anaerobic energy systems [18,19]. Competitive play typically involves oxygen uptake levels surpassing 67% of the $\dot{V}$O_2max_, mean HR reaching approximately 87% of HR_max_, and blood La^−^ averaging around 6.8 mmol·L^−1^ [16,18,20,21]. Our study’s participants exhibited values akin to these observations post-BEST implementation. The recorded 28.42% and 30.38% decreases in sprint and circuit times during the BEST, coupled with escalated RPE and RPMS values afterward, suggest a marked sense of fatigue among the participants. This fatigue and physical exertion hold pivotal significance in determining game outcomes [64], evidenced by prior research indicating that increased heart rate at 80% [65] or 90.7% HR_peak_ [24] led to significant declines in 3-point shot accuracy among elite players. Other studies have delved into biomechanical aspects [66], exploring hip and/or shoulder joint angles and the influence of fatigue on shooting mechanics, showing alterations in joint coordination and neuromuscular aspects [24,25]. Our study aligns with these findings and, importantly, contributes robust evidence, possibly the first of its kind, highlighting the detrimental impact of fatigue induced by a basketball exercise simulation test on 3-point shooting accuracy, release time, and ball entry angle. The implementation of BEST [67] may lead to physical strain, muscle fatigue, and soreness, potentially compromising joint coordination [68,69] and neuromuscular function [70], ultimately impairing technical shooting skills and overall performance [22]. Moreover, physical fatigue induced by high-intensity aerobic exercise can also affect various cognitive aspects of motor performance [71]. The observed POST decreases in 3-point jump shooting accuracy and EA, as well as the increase in RT after a 12 min exercise bout, may be attributed to decreases in the brain’s executive functions and the neuromuscular system’s ability to precisely control movements (for comprehensive reviews, see relevant studies [72,73]). It may also be the case that the central nervous system can process information unaffected by intense aerobic exercise, while the skeletal motor system’s ability is negatively affected during recovery from the effects of physically fatiguing conditions [74]. However, more systematic investigation of the after-effects of exercise-induced fatigue and its impact on the central nervous system’s ability to process information compared to the skeletal motor system’s ability would provide valuable insights.

A systematic review identifies key variables influencing basketball jump shots, including release height correlated with player physical traits like stature and jump ability, release angle linked to the EA [7,8,9], and velocity interlinked with RT. These critical factors intertwine with players’ movement performance, encompassing experience, technical skills, and fitness. In our study, the basketball experience, fatigue indicators, and the correlation coefficient (r) between physiological parameters and differences in 3-point jump shot metrics (i.e., shooting accuracy, RT, and EA) between PRE and POST conditions were consistent across participant groups. However, distinctive variations in anthropometric traits, cardiorespiratory fitness, and lower limb power were evident among groups. Notably, fatigue’s impact from guards to centers more substantially affected shooting accuracy and kinematic parameters. This discrepancy might relate to differences in cardiorespiratory fitness, lower limb power, biomechanical characteristics (e.g., elbow positioning relative to stature during shooting preparation), motor skills, familiarization, and training specifically tailored for 3-point jump shooting among various player positions [14,15,16,18,19,21,75,76]. Yet, in subsequent studies, an in-depth exploration of the identified differences among player positions could involve targeted investigations into the following aspects: cardiorespiratory fitness differences, biomechanical characteristics, motor skill development, and position-specific training programs.

Players with higher cardiorespiratory fitness endure longer fatigue protocols and show better accuracy and consistency in shooting free throws under fatigue [29]. In this study, correlations emerged between participants’ VT_2_ and the changes in RT, EA, and SSs, and among participants’ %HR_max_ during the BEST and the subsequent changes in RT, EA, and SSs. A superior VT_2_ implies enhanced aerobic fitness, allowing high-intensity exercise to be performed for a greater duration (i.e., transition from predominantly aerobic to increased anaerobic energy contribution at higher-intensity exercise). A lower %HR_max_ during the BEST signifies working at a reduced percentage of HR_max_ (i.e., less physiological stress), reflecting better endurance and potentially sustaining shooting accuracy and consistency despite fatigue. Enhanced physical fitness may prompt swifter recovery between physical exertions, preserving shooting precision by lessening fatigue’s impact on muscle function [22,67,68,69]. Although superior cardiorespiratory fitness does not guarantee heightened shooting performance post-fatigue, it may delay fatigue onset or mitigate its effects on shooting mechanics [67,68,69,70]. This sustains muscle function and coordination, refines movement patterns, and supports mental focus during exhausting efforts [77]. While these aspects may contribute to post-fatigue shooting improvement, individual and situational factors require further investigation to comprehend their role in determining shooting accuracy and consistency post-exertion.

### Limitations, Strength, Suggestions for Future Research, and Practical Applications

While this study provided valuable insights, it is essential to acknowledge certain limitations. Firstly, the 12 min duration of the BEST, while inducing physical and physiological fatigue, may not fully replicate the spectrum of fatigue experienced during a full-length basketball game lasting 40 min or more. This difference in duration might elicit distinct physical, physiological, and psychological fatigue responses. Secondly, although participants shot from consistent spots using their dominant hand, the absence of video recording prevented confirmation of identical shooting techniques among participants, potentially impacting the shooting actions’ consistency [5]. Moreover, other kinematic variables, such as various joint positions, velocities, and hip heights, were not measured [78], limiting the depth of our analysis. Thirdly, this study exclusively focused on 3-point jump shots without considering other shot types, and the absence of defensive pressure during these shots might not fully replicate real-game scenarios [4].

However, our study, employing contemporary technology and robust experimental methodologies, corroborates prior observations, specifically concerning 3-point jump shot accuracy, release time, and ball entry angle in relation to player position among high-level athletes. Additionally, the design of the BEST, aligning closely with the activity distribution observed in adult male basketball competitions [16,19,20,55], aimed to replicate realistic game conditions within a controlled laboratory environment. Nevertheless, there remains a need for continued investigation to substantiate and broaden these preliminary outcomes, enriching our comprehension of the impact of basketball-induced fatigue on 3-point shooting accuracy and associated kinematic parameters. It is therefore advisable to explore diverse game-realistic protocols or competitive training drills to assess the effects of fatigue on shooting technical aspects across different shot types and distances, genders, age groups, and skill levels among basketball players.

The current study’s outcomes also have practical applications for basketball coaches and conditioning specialists aiming to enhance technical shooting skills in training sessions. These findings highlight the necessity of integrating technical shooting drills designed to address both aerobic and anaerobic fatigue thresholds. Such targeted training can equip players to manage the rigorous demands of modern basketball, characterized by high-intensity movement and demanding accuracy, particularly evident during 3-point shooting in critical game moments when fatigue is most prevalent. However, the integration of high-intensity technical drills during 3-point shooting sessions should be supervised meticulously to prevent alterations in shooting technique. Understanding the thresholds at which players’ 3-point shooting accuracy, release timing, and ball entry angles are impacted by fatigue levels is crucial. The escalating heart rate in players undergoing physical exertion corresponds to a decline in shooting accuracy and kinematic parameters during fatigue. Hence, designing shooting programs should consider players’ heart rate levels to optimize training effectiveness.

## 5. Conclusions

The findings from this study have broader implications, highlighting the influence of fatigue induced by a basketball exercise simulation test on 3-point shooting accuracy, release time, and ball entry angle in relation to player position. The results emphasize that basketball-induced fatigue negatively affects shooting accuracy, release time, and ball entry angle. These findings also suggest that this fatigue has a more pronounced impact across player positions, notably from guards to centers. Furthermore, this study underscores the association between participants’ cardiorespiratory fitness markers and shooting proficiency post-fatigue induction. Consequently, optimizing training strategies for enhancing jump shot performance is crucial, and there is a need for coaches and players to minimize discrepancies among positions in 3-point shooting accuracy and kinematic parameters while refining shooting techniques.

## Figures and Tables

**Table 1:** Comprehensive analysis of anthropometric and physiological characteristics in guards, forwards and centers groups, and overall (mean ± SD [95%CI]).

Variables	Guards (N = 13)	Forwards (N = 13)	Centers (N = 12)	All Participants (n = 38)
^† ‡ §^ Height (cm)	194.7 ± 2.7 [193.2–196.2]	202.7 ± 2.3 [201.4–203.9]	205.3 ± 0.9 [204.8–205.8]	200.8 ± 5.0 [199.2–202.4]
^† ‡ §^ Body mass (kg)	88.4 ± 3.6 [86.4–90.3]	98.9 ± 4.1 [96.7–101.2]	104.0 ± 2.6 [102.5–105.5]	96.9 ± 7.4 [94.6–99.3]
^† ‡ §^ Body fat (%)	9.4 ± 1.1 [8.8–10.0]	10. 9 ± 1.5 [10.1–11.7]	12.9 ± 1.2 [12.2–13.6]	11.0 ± 1.9 [10.4–11.6]
Age (y)	24.5 ± 3.0 [22.8–26.1]	23.8 ± 2.5 [22.4–25.2]	22.6 ± 2.1 [21.4–23.8]	23.6 ± 2.7 [22.8–24.5]
Experience practicing basketball (y)	13.4 ± 3.3 [11.6–15.2]	13.1 ± 2.7 [11.6–14.6]	11.9 ± 1.9 [10.9–13.0]	12.8 ± 2.7 [11.9–13.7]
Active in basketball competition (y)	12.5 ± 3.0 [10.8–14.1]	11.8 ± 2.5 [10.4–13.2]	10.6 ± 2.1 [9.4–11.8]	11.6 ± 2.7 [10.8–12.5]
Physical exercise training (h·wk^−1^)	4.2 ± 0.9 [3.7–4.7]	3.8 ± 1.1 [3.2–4.4]	3.6 ± 0.8 [3.2–4.1]	3.9 ± 1.0 [3.6–4.2]
Basketball related training (h·wk^−1^)	10.8 ± 1.4 [10.1–11.6]	10.3 ± 0.9 [9.8–10.8]	10.8 ± 1.1 [10.2–11.5]	10.7 ± 1.2 [10.3–11.0]
^† §^ $\dot{V}$O_2max_ (mL·kg^−1^·min^−1^)	60.9 ± 2.5 [59.6–62.3]	59.4 ± 2.2 [58.2–60.6]	57.1 ± 1.6 [56.2–58.0]	59.2 ± 2.6 [58.3–60.0]
^† ‡^ VT_2_ (%$\dot{V}$O_2max_)	81.1 ± 1.4 [80.3–81.9]	77.1 ± 2.1 [76.0–78.3]	75.5 ± 2.2 [74.3–76.7]	78.0 ± 3.0 [77.0–78.9]
HR_max_ (b·min^−1^)	195.7 ± 2.3 [194.4–196.9]	196.0 ± 1.8 [195.0–197.0]	197.0 ± 1.2 [196.3–197.7]	196.2 ± 1.9 [195.6–196.8]
^† ‡ §^ CMJ height (cm)	51.7 ± 2.5 [50.3–53.0]	46.5 ± 1.7 [45.6–47.4]	42.0 ± 1.0 [41.4–42.6]	46.8 ± 4.4 [45.4–48.2]

^†^ Significant difference between guards and centers groups at *p* ≤ 0.05. ^‡^ Significant difference between guards and forwards groups at *p* ≤ 0.05. ^§^ Significant difference between forwards and centers groups at *p* ≤ 0.05. Abbreviations: BF, body fat; BM, body mass; CMJ, countermovement jump; HR_max_, maximum heart rate; M, mean; n, sample size of the study; N, sample size of the groups; SD, standard deviation; VT_2_, second ventilatory threshold; $\dot{V}$O_2max_, maximum oxygen uptake; and [95%CI], 95% confidence interval.

**Table 2:** The M ± SD [95%CI] of ST decrease, CT decrease, and mean HR during the BEST, and La^−^, RPE, and RPMS after the BEST in guards, forwards, and centers groups, and overall.

Dependent Variables	Guards (N = 13)	Forwards (N = 13)	Centers (N = 12)	All Participants (n = 38)
^† §^ ST decrease (%)	27.8 ± 0.6 [27.5–28.2]	28.2 ± 0.7 [27.8–28.5]	29.3 ± 1.4 [28.5–30.1]	28.4 ± 1.1 [28.1–28.8]
CT decrease (%)	30.6 ± 2.5 [29.2–31.9]	30.2 ± 2.7 [28.7–31.7]	30.4 ± 2.5 [29.0–31.8]	30.4 ± 2.5 [29.6–31.2]
HR (%HR_max_)	88.8 ± 2.3 [87.6–90.1]	90.7 ± 3.6 [88.7–92.7]	89.6 ± 3.1 [87.8–91.3]	89.7 ± 3.1 [88.7–90.7]
La^−^ (mmol·L^−1^)	6.5 ± 0.9 [6.0–7.0]	5.8 ± 1.2 [5.1–6.4]	6.0 ± 0.7 [5.6–6.4]	6.1 ± 1.0 [5.8–6.4]
RPE (Borg scale, 6–20)	4.0 ± 0.8 [3.55–4.4]	3.9 ± 0.6 [3.6–4.3]	3.8 ± 0.8 [3.4–4.3]	3.9 ± 0.7 [3.7–4.2]
RPMS (Likert scale, 0–10)	13.2 ± 1.1 [12.6–13.8]	12.9 ± 0.9 [12.4–13.4]	13.5 ± 1.2 [12.8–14.2]	13.2 ± 1.1 [12.9–13.6]

^†^ Significant difference between guards and centers groups at *p* ≤ 0.05. ^§^ Significant difference between forwards and centers groups at *p* ≤ 0.05. Abbreviations: BEST, basketball exercise simulation test; CT, circuit time; HR, heart rate; HR_max_, maximum heart rate; La^−^, blood lactate; M, mean; n, sample size of the study; N, sample size of the groups; RPE, subjective rate of perceived exertion; RPMS, subjective rate of muscle soreness; SD, standard deviation; ST, sprint time; and [95%CI], 95% confidence interval.

**Table 3:** The M ± SD [95%CI] of mean 3-point jump shot release time, ball’s entry angle into the hoop, and success of guards, forwards, and centers groups, and overall in two conditions, PRE and POST.

	Guards (N = 13)		Forwards (N = 13)		Centers (N = 12)		All Participants (n = 38)	
Dependent Variables	PRE	POST	PRE	POST	PRE	POST	PRE	POST
RT (s)	* 0.6 ± 0.0 [0.5–0.6]	0.7 ± 0.0 [0.7–0.7]	* 0.7 ± 0.1 [0.7–0.7]	0.8 ± 0.0 [0.8–0.9]	* 0.8 ± 0.1 [0.8–0.9]	0.9 ± 0.1 [0.9–1.0]	* 0.7 ± 0.1 [0.7–0.7]	0.8 ± 0.1 [0.8–0.9]
EA (°)	* 45.2 ± 0.9 [44.7–45.7]	43.4 ± 1.1 [42.8–44.1]	* 43.3 ± 1.4 [42.6–44.1]	42.0 ± 1.0 [41.4–42.5]	* 41.8 ± 0.8 [41.4–42.3]	40.4 ± 1.0 [39.8–40.9]	* 43.5 ± 1.7 [43.0–44.1]	42.0 ± 1.6 [41.4–42.5]
SSs (frequency)	* 7.8 ± 0.4 [7.5–8.0]	6.6 ± 0.5 [6.3–6.9]	* 7.1 ± 0.5 [6.8–7.4]	5.9 ± 0.6 [5.6–6.3]	* 6.3 ± 0.5 [6.0–6.6]	5.1 ± 0.7 [4.7–5.5]	* 7.1 ± 0.8 [6.9–7.3]	5.9 ± 0.9 [5.6–6.2]
SSR (%)	* 51.8 ± 2.9 [50.2–53.4]	44.1 ± 3.4 [42.3–45.9]	* 47.7 ± 3.7 [45.7–49.7]	39.5 ± 4.3 [37.2–41.8]	* 42.2 ± 3.3 [40.4–44.1]	33.9 ± 4.5 [31.4–36.4]	* 47.4 ± 5.1 [45.7–49.0]	39.3 ± 5.8 [37.5–41.1]

* Significant difference between conditions at *p* ≤ 0.05. Abbreviations: BEST, basketball exercise simulation test; EA, entry angle into the hoop; M, mean; n, sample size of the study; N, sample size of the groups; POST, condition after the BEST; PRE, condition prior to the BEST; RT, jump shot release time; SD, standard deviation; SSR, shooting success rate; SSs, successful shots; and [95%CI], 95% confidence interval.

**Table 4:** Three-point jump shot release time, ball’s entry angle into the hoop, and successful shots estimates are presented as M ± SE [95%CI] under the POST condition according to the participant groups, adjusted for the covariate values of the PRE condition.

Dependent Variables	No.	Group, N	Estimated Value in the POST Condition	Significance
^¶^ RT (s)	1	Guards, 13	0.7 ± 0.0 [0.6–0.8]	* 1 < 2, 3
	2	Forwards, 13	0.8 ± 0.0 [0.8–0.9]	* 2 < 3
	3	Centers, 12	0.9 ± 0.0 [0.9–1.0]	
^#^ EA (°)	1	Guards, 13	43.4 ± 0.4 [42.6–44.2]	* 1 > 2, 3
	2	Forwards, 13	42.0 ± 0.3 [41.4–42.6]	* 2 > 3
	3	Centers, 12	40.4 ± 0.4 [39.6–41.2]	
^‖^ SSs (frequency)	1	Guards, 13	6.6 ± 0.2 [6.2–7.1]	* 1 > 2, 3
	2	Forwards, 13	5.9 ± 0.2 [5.6–6.3]	* 2 > 3
	3	Centers, 12	5.0 ± 0.2 [4.6–5.5]	

^¶^ The value of 0.6921 s (PRE value) was used to evaluate covariates in the model. ^#^ The value of 43.5026° (PRE value) was used to evaluate covariates in the model. ^‖^ The value of 7.11 shots (PRE value) was used to evaluate covariates in the model. * *p* ≤ 0.05, significant effect of BEST on groups. Abbreviations: BEST, basketball exercise simulation test; EA, entry angle into the hoop; M, mean; N, sample size of the groups; POST, condition after the BEST; PRE, condition prior to the BEST; RT, jump shot release time; SE, standard error; SSs, successful shots; and [95%CI], 95% confidence interval.

**Table 5:** Pearson correlation coefficient (r) between physiological parameters and the observed differences in 3-point jump shot release time, ball’s entry angle into the hoop, and shooting accuracy between the POST and PRE conditions within the studied participant sample (n = 38).

Dependent Variables	$\dot{V}$O_2max_ (mL·kg^−1^·min^−1^)	VT_2_ (%$\dot{V}$O_2max_)	HR (%HR_max_)
RT (s)	0.13	* –0.50	* 0.75
EA (°)	–0.20	* 0.48	* –0.64
SSs (frequency)	–0.09	* 0.50	* –0.68

* Significant correlation at *p* < 0.05. Abbreviations: BEST, basketball exercise simulation test; EA, entry angle into the hoop; HR, mean heart rate during the BEST; HR_max_, maximum heart rate; n, sample size of the study; POST, condition after the BEST; PRE, condition prior to the BEST; RT, jump shot release time; SSR, shooting success rate; SSs, successful shots; $\dot{V}$O_2max_, maximum oxygen uptake; VT_2_, second ventilatory threshold.

## Data Availability

The raw data supporting the conclusions of this article will be made available by the corresponding author upon reasonable request once all relevant substudies are reported and completed. The study protocol, data dictionary, and statistical analysis plan can also be made available by the corresponding author upon request.

## Supplementary Materials

The following supporting information can be downloaded at: https://www.mdpi.com/article/10.3390/sports12030063/s1, File: Supplementary Methods.

## References

[1] FIBA (2022). International Basketball Federation Official Basketball Rules 2022.

[2] Theodorou A.S., Rizou H.P., Zacharakis E., Ktistakis I., Bekris E., Panoutsakopoulos V., Strouzas P., Bourdas D.I., Kostopoulos N. (2022). Pivot Step Jump: A New Test for Evaluating Jumping Ability in Young Basketball Players. J. Funct. Morphol. Kinesiol..

[3] Tang W.T., Shung H.M. (2005). Relationship between isokinetic strength and shooting accuracy at different shooting ranges in Taiwanese elite high school basketball players. Isokinet. Exerc. Sci..

[4] Rojas F.J., Oña A., Gutierrez M., Cepero M. (2000). Kinematic adjustments in the basketball jump shot against an opponent. Ergonomics.

[5] Knudson D. (1993). Biomechanics of the Basketball Jump Shot—Six Key Teaching Points. J. Phys. Educ. Recreat. Danc..

[6] Okazaki V.H.A., Rodacki A.L.F., Satern M.N. (2015). A review on the basketball jump shot. Sports Biomech..

[7] Aksović N., Bjelica B., D’Onofrio R., Milanović F., Nikolić D., Pržulj R. (2022). Kinematic Analysis of Basketball Jump Shot. Ital. J. Sports Rehabil. Posturol..

[8] Fontanella J.J. (2006). The Physics of Basketball.

[9] Brancazio P.J. (1981). Physics of basketball. Am. J. Phys..

[10] Okazaki V.H.A., Rodacki A.L.F. (2012). Increased distance of shooting on basketball jump shot. J. Sport. Sci. Med..

[11] Crowley M.J., King K. (2010). Monitoring of Physical Training Events.

[12] Dobovičnik L., Saša J., Zovko V., Erčulj F. (2015). Determination of the optimal certain kinematic parameters in basketball three-point shooting using the 94fifty technology. Phys. Cult..

[13] Stojanović E., Radenković M., Bubanj S., Stanković R. (2019). Kinematic parameters of the jump shot in elite male basketball players. Phys. Educ. Sport.

[14] Zacharakis E.D., Bourdas D.I., Kotsifa M.I., Bekris E.M., Velentza E.T., Kostopoulos N.I. (2020). Effect of balance and proprioceptive training on balancing and technical skills in 13–14-year-old youth basketball players. J. Phys. Educ. Sport.

[15] Zacharakis E., Souglis A., Bourdas D., Gioldasis A., Apostolidis N., Kostopoulos N. (2021). The relationship between physical and technical performance characteristics of young soccer and basketball players: A comparison between two sports. Gazz. Medica Ital. Arch. Per Le Sci. Mediche.

[16] Stojanović E., Stojiljković N., Scanlan A.T., Dalbo V.J., Berkelmans D.M., Milanović Z. (2018). The Activity Demands and Physiological Responses Encountered During Basketball Match-Play: A Systematic Review. Sports Med..

[17] Bourdas D.I., Mitrousis I., Zacharakis E.D., Travlos A.K. (2022). Home-audience advantage in basketball: Evidence from a natural experiment in Euro League games during the 2019–2021 COVID-19 era. J. Phys. Educ. Sport.

[18] Bourdas D.I., Zacharakis E.D., Travlos A.K., Souglis A. (2021). Return to Basketball Play Following COVID-19 Lockdown. Sports.

[19] Petway A.J., Freitas T.T., Calleja-González J., Leal D.M., Alcaraz P.E. (2020). Training load and match-play demands in basketball based on competition level: A systematic review. PLoS ONE.

[20] Paulauskas R., Kamarauskas P., Nekriošius R., Bigwood N.M. (2020). Physical and Physiological Response to Different Modes of Repeated Sprint Exercises in Basketball Players. J. Hum. Kinet..

[21] Narazaki K., Berg K., Stergiou N., Chen B. (2009). Physiological demands of competitive basketball. Scand. J. Med. Sci. Sports.

[22] Yang C., Leitkam S., Coté J.N. (2019). Effects of different fatigue locations on upper body kinematics and inter-joint coordination in a repetitive pointing task. PLoS ONE.

[23] Erčulj F., Supej M. (2006). The Impact of Fatigue on Jump Shot Height and Accuracy Over a Longer Shooting Distance in Basketball. Balt. J. Sport Health Sci..

[24] Erculj F., Supej M. (2009). Impact of fatigue on the position of the release arm and shoulder girdle over a longer shooting distance for an elite basketball player. J. Strength Cond. Res..

[25] Slawinski J., Poli J. Effect of fatigue on basketball three points shot kinematics. Proceedings of the 33 International Conference of Biomechanics in Sports.

[26] Marcolin G., Camazzola N., Panizzolo F.A., Grigoletto D., Paoli A. (2018). Different intensities of basketball drills affect jump shot accuracy of expert and junior players. PeerJ.

[27] Rupcic T., Knjaz D., Bakovic M., Devrnja A., Matkovic B.R. (2015). Impact of fatigue on accuracy and changes in certain kinematic parameters during shooting in basketball. Hrvat. Sport. Vjesn..

[28] Mulazimoglu O., Yanar S., Evcil A.T., Duvan A. (2017). Examining the Effect of Fatigue on Shooting Accuracy in Young Basketball Players. Int. J. Soc. Humanit. Sci. Res..

[29] Leigh S., Rolfe B., Konz S. (2019). Cardiorespiratory Fitness Alleviates the Effect of Fatigue on Basketball Free Throw Shooting Performance. Proceedings of the 37th International Society of Biomechanics in Sport Conference.

[30] Freitas L. (2021). Shot distribution in the NBA: Did we see when 3-point shots became popular?. Ger. J. Exerc. Sport Res..

[31] Rupčić T., Feng L., Matković B.R., Knjaz D., Dukarić V., Baković M., Matković A., Svoboda I., Vavaček M., Garafolić H. (2020). The impact of progressive physiological loads on angular velocities during shooting in basketball-case study. Acta Kinesiol..

[32] Slawinski J., Louis J., Poli J., Tiollier E., Khazoom C., Dinu D. (2018). The Effects of Repeated Sprints on the Kinematics of 3-Point Shooting in Basketball. J. Hum. Kinet..

[33] Ardigò L.P., Kuvacic G., Iacono A.D., Dascanio G., Padulo J. (2018). Effect of heart rate on basketball three-point shot accuracy. Front. Physiol..

[34] World Health Organization (2016). Health Behaviour in School-Aged Children Study: Physical Activity in Adolescents Key Facts and Figures.

[35] Bourdas D.I., Zacharakis E.D. (2020). Impact of COVID-19 Lockdown on Physical Activity in a Sample of Greek Adults. Sports.

[36] Warburton D., Jamnik V., Bredin S., Gledhill N. (2018). The 2018 Physical Activity Readiness Questionnaire for Everyone (PAR-Q+) and electronic Physical Activity Readiness Medical Examination (ePARmed-X+). Health Fit. J. Can..

[37] Bourdas D.I., Zacharakis E.D., Travlos A.K., Souglis A., Georgali T.I., Gofas D.C., Ktistakis I.E., Deltsidou A. (2021). Impact of lockdown on smoking and sleeping in the early COVID-19 presence: Datasets of Greek Adults sample. Data Br..

[38] World Medical Association (2013). Declaration of Helsinki, Ethical Principles for Scientific Requirements and Research Protocols. Bull. World Health Organ..

[39] Jackson A.S., Pollock M.L. (1978). Generalized equations for predicting body density of men. Br. J. Nutr..

[40] Clemente F.M., Nikolaidis P.T., Rosemann T., Knechtle B. (2019). Dose-response relationship between external load variables, body composition, and fitness variables in professional soccer players. Front. Physiol..

[41] Riebe D., American College of Sports Medicine (2018). ACSM’s Guidelines for Exercise Testing and Prescription.

[42] Borg G.A.V. (1982). Psychophysical bases of perceived exertion. Med. Sci. Sports Exerc..

[43] Cerezuela-Espejo V., Courel-Ibáñez J., Morán-Navarro R., Martínez-Cava A., Pallarés J.G. (2018). The relationship between lactate and ventilatory thresholds in runners: Validity and reliability of exercise test performance parameters. Front. Physiol..

[44] Wasserman K., Whipp B.J., Koyal S.N., Beaver W.L. (1973). Anaerobic threshold and respiratory gas exchange during exercise. J. Appl. Physiol..

[45] Gaskill S.E., Ruby B.C., Walker A.V.A.J., Sanchez O.A., Serfass R.C., Leon A.S. (2001). Validity and reliability of combining three methods to determine ventilatory threshold. Med. Sci. Sports Exerc..

[46] Scanlan A.T., Dascombe B.J., Reaburn P.R.J. (2014). Development of the basketball exercise simulation test: A match-specific basketball fitness test. J. Hum. Sport Exerc..

[47] Bourdas D.I., Travlos A.K., Souglis A., Stavropoulou G., Zacharakis E., Gofas D.C., Bakirtzoglou P. (2024). Effects of a Singular Dose of Mangiferin—Quercetin Supplementation on Basketball Performance: A Double-Blind Crossover Study of High-Level Male Players. Nutrients.

[48] Calleja-González J., Terrados N., Mielgo-Ayuso J., Delextrat A., Jukic I., Vaquera A., Torres L., Schelling X., Stojanovic M., Ostojic S.M. (2016). Evidence-based post-exercise recovery strategies in basketball. Phys. Sportsmed..

[49] Mihajlovic M., Cabarkapa D., Cabarkapa D.V., Philipp N.M., Fry A.C. (2023). Recovery Methods in Basketball: A Systematic Review. Sports.

[50] Bourdas D.I., Souglis A., Zacharakis E.D., Geladas N.D., Travlos A.K. (2021). Meta-Analysis of Carbohydrate Solution Intake during Prolonged Exercise in Adults: From the Last 45+ Years’ Perspective. Nutrients.

[51] Deltsidou A., Zarikas V., Mastrogiannis D., Kapreli E., Bourdas D., Raftopoulos V., Noula M., Lykeridou K. (2020). Data on advanced glycation end-products concentrations and haemodynamic parameters following caffeine and nicotine consumption in nursing students. Data Br..

[52] Havenetidis K., Bourdas D. (2003). Creatine supplementation: Effects on urinary excretion and anaerobic performance. J. Sports Med. Phys. Fit..

[53] Souglis A., Bourdas D.I., Gioldasis A., Ispirlidis I., Philippou A., Zacharakis E., Apostoldis A., Efthymiou G., Travlos A.K. (2023). Time Course of Performance Indexes, Oxidative Stress, Inflammation, and Muscle Damage Markers after a Female Futsal Match. Sports.

[54] Glaister M., Howatson G., Pattison J.R., McInnes G. (2008). The reliability and validity of fatigue measures during multiple-sprint work: An issue revisited. J. Strength Cond. Res..

[55] Scanlan A., Dascombe B., Reaburn P. (2011). A comparison of the activity demands of elite and sub-elite Australian men’s basketball competition. J. Sports Sci..

[56] Rupčić T., Antekolović L., Knjaz D., Matković B., Cigrovski V., Zvonař M., Sajdlová Z. (2015). Reliability analysis of the 94 fifty smart sensor basketball. Proceedings of the 10th International Conference on Kinanthropology “Sport and Quality of Life”.

[57] Abdelrasoul E., Mahmoud I., Stergiou P., Katz L. (2015). The accuracy of a real time sensor in an instrumented basketball. Procedia Eng..

[58] Walther L.H., Zegers F., Nybo M., Mogensen C.B., Christensen E.F., Lassen A.T., Mikkelsen S. (2022). Accuracy of a point-of-care blood lactate measurement device in a prehospital setting. J. Clin. Monit. Comput..

[59] Nova Biomedical (2011). Nova StatStrip Xpress Lactate Hospital Meter Instructions for Use Manual.

[60] Nosaka K., Sacco P., Mawatari K. (2006). Effects of amino acid supplementation on muscle soreness and damage. Int. J. Sport Nutr. Exerc. Metab..

[61] Keppel G., Wickens T.D. (2004). Design and Analysis: A Researcher’s Handbook.

[62] Cohen J. (1988). Statistical Power Analysis for the Behavioral Sciences.

[63] Bland J.M., Altman D.G. (1986). Statistical methods for assessing agreement between two methods of clinical measurement. Lancet.

[64] Lyons M., Al-Nakeeb Y., Nevill A. (2006). The impact of moderate and high intensity total body fatigue on passing accuracy in expert and novice basketball players. J. Sports Sci. Med..

[65] Padulo J., Attene G., Migliaccio G.M., Cuzzolin F., Vando S., Ardigò L.P. (2015). Metabolic optimisation of the basketball free throw. J. Sports Sci..

[66] Li F., Knjaz D., Rupčić T. (2021). Influence of fatigue on some kinematic parameters of basketball passing. Int. J. Environ. Res. Public Health.

[67] Sterner R.L., Pincivero D.M., Lephart S.M. (1998). The effects of muscular fatigue on shoulder proprioception. Clin. J. Sport Med..

[68] Hiemstra L.A., Lo I.K.Y., Fowler P.J. (2001). Effect of fatigue on knee proprioception: Implications for dynamic stabilization. J. Orthop. Sports Phys. Ther..

[69] Skinner H.B., Wyatt M.P., Hodgdon J.A., Conard D.W., Barrack R.L. (1986). Effect of fatigue on joint position sense of the knee. J. Orthop. Res..

[70] Chen W., Lo S.-L., Lee Y.-K., Wang J.-S., Shiang T.-Y. (2005). Effects of Upper Extremity Fatigue on Basketball Shooting Accuracy. Proceedings of the 23 International Symposium on Biomechanics in Sports.

[71] Sudo M., Costello J.T., McMorris T., Ando S. (2022). The effects of acute high-intensity aerobic exercise on cognitive performance: A structured narrative review. Front. Behav. Neurosci..

[72] McMorris T. (2021). The acute exercise-cognition interaction: From the catecholamines hypothesis to an interoception model. Int. J. Psychophysiol..

[73] Tempest G.D., Davranche K., Brisswalter J., Perrey S., Radel R. (2017). The differential effects of prolonged exercise upon executive function and cerebral oxygenation. Brain Cogn..

[74] Travlos A.K., Marisi D.Q. (1995). Information processing and concentration as a function of fitness level and exercise-induced activation to exhaustion. Percept. Mot. Ski..

[75] Cabarkapa D., Fry A.C., Cabarkapa D.V., Myers C.A., Jones G.T., Philipp N.M., Yu D., Deane M.A. (2022). Differences in Biomechanical Characteristics between Made and Missed Jump Shots in Male Basketball Players. Biomechanics.

[76] StatMuse NBA League Average 3 Point Percentage by Position. https://www.statmuse.com/nba/ask/nba-league-average-3-point-percentage-by-position.

[77] Kohl H.W., Cook H.D., Committee on Physical Activity and Physical Education in the School Environment, Food and Nutrition Board, Institute of Medicine (2013). Educating the Student Body: Taking Physical Activity and Physical Education to School.

[78] França C., Gouveia É.R., Silva M.J.C., Gomes B.B. (2022). A kinematic analysis of the basketball shot performance: Impact of distance variation to the basket. Acta Bioeng. Biomech..

