# Supplementary material for: Basketball Fatigue Impact on Kinematic Parameters and 3-Point Shooting Accuracy: Insights across Players’ Positions and Cardiorespiratory Fitness Associations of High-Level Players
Source: Sports (Basel). 2024 Feb 20;12(3):63. doi: 10.3390/sports12030063 (PMC10974731; doi:10.3390/sports12030063)
Supplement: Supplementary file 1 [file sports-12-00063-s001.zip › sports-2822543-supplementary.pdf]

Supplementary Methods

Formulas to calculate body fat [1]

% body fat = $[(4.95 \cdot Db^{-1}) - 4.5] \cdot 100$

$Db = 1.112 - 0.00043499 \cdot x_1 + 0.00000055 \cdot x_1^2 - 0.00028826 \cdot x_2$

x_1 = sum of chest, axilla, triceps, subscapula, abdomen, suprailium and front thigh skinfolds

x_2 = age (year)

Countermovement jump height test protocol

The countermovement jump (CMJ) height test was conducted after a standardized 5 min warm up at 60–70% intensity on a Monark leg cycle ergometer (894E®, Monark, Varberg, Sweden), predefined dynamic stretches, and 3-5 practice jumps [2]. Next, the participants from a standing position with their knees slightly bent and their arms hanging loosely at their sides, quickly lowers their body by bending their knees and hips until their thighs are parallel to the ground and then explosively extend their legs and hips, pushing off the ground as hard as possible while simultaneously swinging their arms overhead and continue to extend their legs and arms as they reach their maximum height.

References

1. Jackson, A.S.; Pollock, M.L. Generalized equations for predicting body density of men. *Br. J. Nutr.* **1978**, *40*, 497–504, doi:10.1079/bjn19780152.
2. Clemente, F.M.; Nikolaidis, P.T.; Rosemann, T.; Knechtle, B. Dose-response relationship between external load variables, body composition, and fitness variables in professional soccer players. *Front. Physiol.* **2019**, *10*, 443, doi:10.3389/fphys.2019.00443.
